# A Rare Case of Giant Occipital Encephalocele With Thoracic Myelomeningocele: An Anesthetic Conundrum

**DOI:** 10.7759/cureus.29602

**Published:** 2022-09-26

**Authors:** Jitendra V Kalbande, Ketki D Deotale, Subrata K Singha, Habib Md R Karim, Rashmi Dubey

**Affiliations:** 1 Anesthesiology, Critical Care and Pain Medicine, All India Institute of Medical Sciences, Raipur, Raipur, IND

**Keywords:** paediatric neurosurgery, paediatric anesthesia, encephalocele with myelomeningocele, neonates, multiple neural tube defect, myelomeningocele, encephalocele, difficult airway

## Abstract

Encephalocele and myelomeningocele are congenital defects in the cranium and spine with herniation of contents into an extracranial and extraspinal sac, respectively. The occurrence of encephalocele and myelomeningocele in the same patient has rarely been described in the literature. The anesthetic management of such cases is associated with multiple challenges, which include difficulty in securing the airway, prone positioning, blood loss, electrolyte imbalance, hypothermia, cardiorespiratory disturbances, and perioperative care. The main aims are, to prevent hemodynamic fluctuations and excessive pressure on the sac to avoid premature rupture and manage a possible difficult airway due to the head and neck mass. We report such a rare case to highlight and share our experiences faced during perioperative management of a giant vascular occipital encephalocele with impending rupture and thoracic myelomeningocele requiring surgical excision and repair. Previous similar case reports were also reviewed, and potential perioperative complications were discussed.

## Introduction

Neural tube defects (NTDs) are common congenital anomalies involving any part of the vertebral column. Encephaloceles are congenital herniations of intracranial contents into an extracranial sac through a skull defect and occur in one in 5,000 live births, more frequently in the occipital region [1]. Myelomeningocele (MMC) is the most common NTD, compatible with life, with an incidence of 0.44-1 per 1,000 live births [2]. In MMC, both neural and meningeal elements are exposed via a midline bony defect in the spine. Multiple neural tube abnormalities in the same patient are infrequent. Further, it is highly uncommon to have encephalocele with vascular connection with the brain and their association with MMC [3]. The age of the patient population, i.e., neonates, their anatomic-physiological changes, hemodynamic fluctuations, coupled with encephalocele with MMC, is a perioperative challenge requiring scrupulous conduction of anesthesia. In this manuscript, we describe the problems faced and preventive measures considered for anesthetic management of such a rare case and discuss them in light of existing literature.

## Case presentation

A 15-day-old male neonate weighing 3.2 kg presented with two swellings: one giant cystic swelling, larger than the head in the occipital region of the skull, and another medium-sized swelling in the thoracic area of the spine since birth. The child was scheduled for surgical excision and repair. The swellings were gradually increasing in size since birth. The neonate was second-born by normal vaginal delivery, out of non-consanguineous marriage, birth weight being 2.7 kg at a peripheral hospital. He was accepting breastfeeds well since birth, with normal for age bladder and bowel habits. On the eighth day of life, the infant developed a yellowish discoloration of skin and eyes, which was diagnosed as physiological jaundice and managed conservatively.

**Figure 1:**
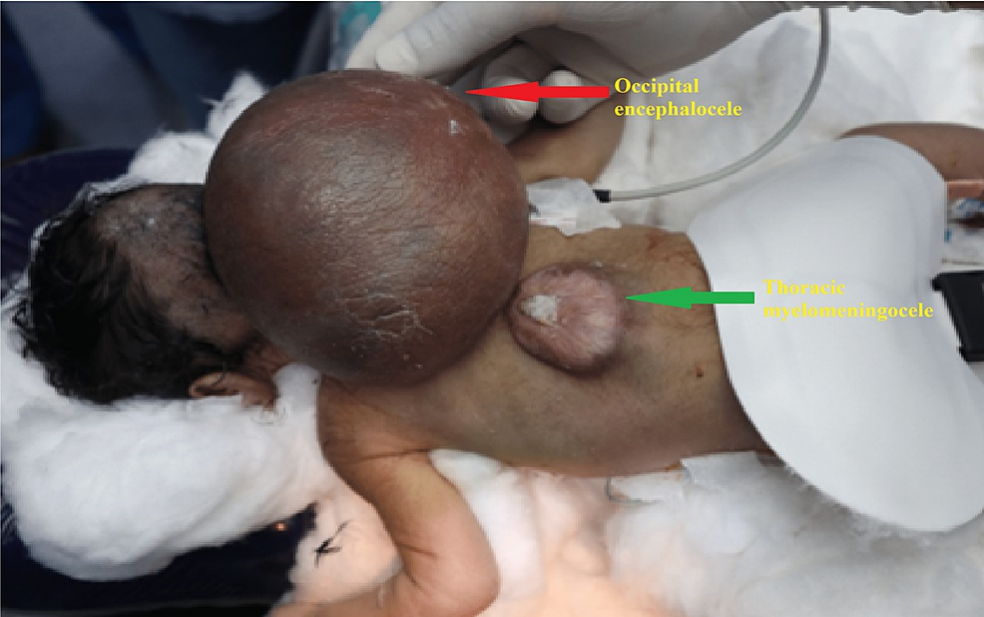
Occipital encephalocele with thoracic MMC in the neonate in a prone position. Red arrow pointing towards occipital encephalocele and green arrow towards thoracic MMC. MMC - Myelomeningocele

On examination, the baby was afebrile, active, alert, and preferred to lie in the lateral position. Spontaneous eye opening was present, pupils mid-dilated and equally reacting to light. Spontaneously moving the upper limbs with normal tone and power, i.e., moving b/l upper limb against antigravity and resistance spontaneously. However, there was a weakness in bilateral lower limbs with decreased movements, tone, and power, i.e., movements against antigravity seen. No signs of meningeal irritation or convulsions were observed. The occipital swelling was 15 cm × 12 cm × 10 cm in size. It was globular, cystic, fluctuating, non-tender, protruding from the occipital region of the skull, and impending to rupture at any time. The skin overlying the mass was red and glistening. Another swelling was present in the lower thoracic region, measuring 6 cm x 4 cm in size, which was partially epithelialized (Figure 1).

**Figure 2:**
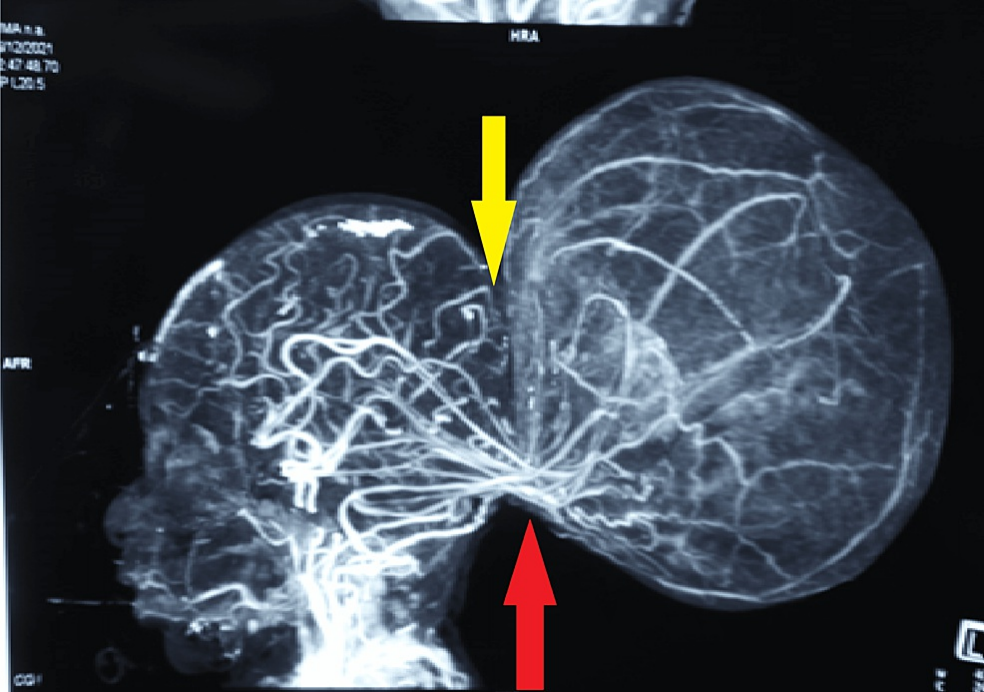
CT angiogram of the brain vessels. Yellow arrow showing a defect in the occipital bone. Red arrow showing intracranial vessels entering into the sac and looping out of the sac. CT - Computed tomography.

Preoperative magnetic resonance imaging (MRI) showed a sac measuring 11cm x 6 cm x 7 cm herniating through the defect measuring 1.5 cm x 0.9 cm, along with herniation of the blood vessels, cerebellum, and fourth ventricle, with peripheral blooming within the sac suggestive of hemorrhage. Agenesis of the corpus callosum was also noted. In addition, a posterior arch defect was noted from the D10 to L5 vertebral level, with herniation of the spinal cord and cerebrospinal fluid (CSF) filled meninges. Computed tomography (CT) angiogram revealed communicating blood vessels between the brain and the occipital encephalocele, intracranial vessels entering the sac and looping out of the sac to supply normal brain parenchyma, indicating the vascular nature of the swelling (Figure 2). A diagnosis of occipital encephalocele with impending rupture and thoracic unruptured MMC was made, and the patient was scheduled for surgery for a threatened rupture of the encephalocele. The baby's respiratory and cardiovascular examinations were unremarkable. The preoperative hematological and biochemical results were within normal limits.

**Figure 3:**
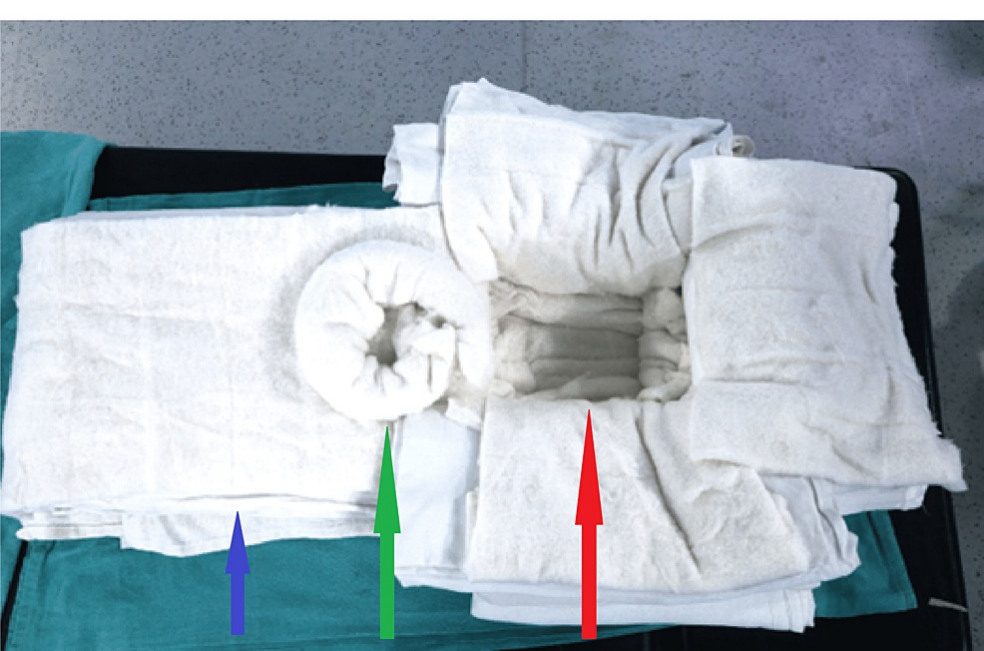
Arrangement for positioning neonate for intubation. Blue arrow pointing to big surgical drapes and cotton. A green arrow points to the doughnut ring cushion to support thoracic MMC. Red arrow pointing support for occipital encephalocele. MMC - Myelomeningocele

**Figure 4:**
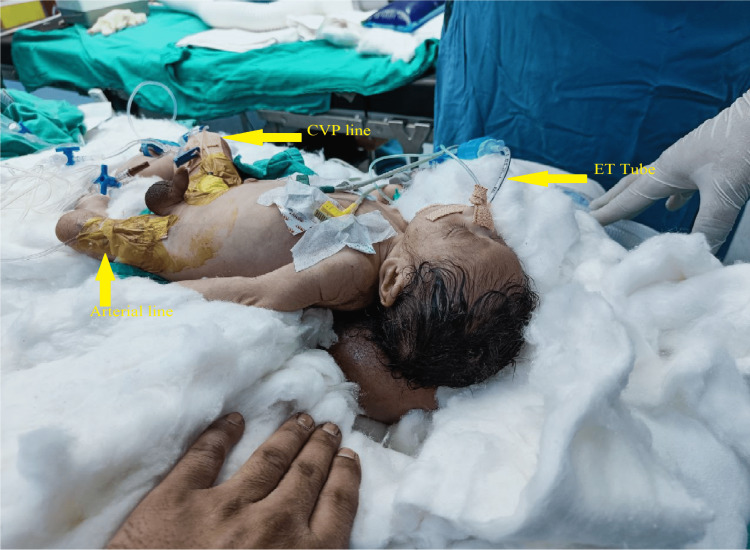
Arrangement of the "four stacks" supporting and protecting both swellings. ET - Endotracheal tube, CVP - Central venous pressure.

The baby fasted for breast milk for four hours, and maintenance fluid (i.e., ringer lactate) was started at 10mL/hour. It was difficult to optimally position the head in the supine position due to the giant occipital encephalocele making bag-mask ventilation and airway management tricky and challenging. To stabilize and optimize the head position for airway management in the supine position, improvised head supports were made using surgical drapes and cotton pads. The surgical drapes and cotton pads were stacked on top of each other, and four such stacks were made. The superficial layer of each stack was covered with a cotton pad to provide a cushioning effect against swelling. One of the stacks was placed at the head end, one at the foot end, and two laterally to achieve the configuration shown in the picture (Figure 3). This way, a square, recessed space was created, covered with cotton to accommodate the encephalocele with a cushioning effect and to ensure that other blocks supported the head to maintain a neutral supine position. As a result, the huge cystic swelling rested on a soft surface without excessive pressure applied to the swelling. At the foot end stack, we made a “doughnut” ring cushion to the size of the thoracic MMC with a cotton roll to protect and support the defect in the thoracic region. This arrangement allowed conventional supine positioning and some dynamic movement of the head and neck during mask ventilation and direct laryngoscopy (Figure 4).

The temperature was optimized in operation theatre (OT) to nearly 26-27 degrees; the difficult airway cart was kept ready. American Society of Anesthesiologists standard monitors (electrocardiogram, pulse oximetry, blood pressure, temperature) were connected. The baby was premedicated with glycopyrrolate 4 mcg/kg and fentanyl 1 mcg/kg intravenously (iv), and general anesthesia (GA) was induced with sevoflurane and propofol (1 mg/kg) in the supine posture. After confirming the feasibility of bag-mask ventilation, muscle relaxation was achieved with intravenous succinylcholine 6 mg. The trachea was intubated successfully with a 3-mm uncuffed endotracheal tube on the first attempt. After confirming the correct endotracheal tube position, the tube was fixed, and throat packing was done. Considering the vascularity of the swelling, the left femoral artery was cannulated with a 22G catheter for invasive blood pressure and arterial blood gas (ABG) monitoring, while an ultrasound-guided, 4.5 Fr triple lumen central venous pressure (CVP) catheter was secured in the right femoral vein. The patient was positioned prone for surgery; bilateral air entry re-confirmed. Intraoperatively electrocardiogram, pulse oximetry, capnography, invasive blood pressure, skin temperature, urine output, blood sugar, and ABG monitoring was done.

Before skin incision antibiotics ceftraixone 50 mg/kg (intravenous) was given. Injection dexamethasone 0.1 mg/kg was given for brain edema due to surgical handling. Considering the possibility of postoperative seizures, levetiracetam 10mg/kg was administered and continued postoperatively.

During surgery, the presence of blood vessels in the sac complicated the resection, resulting in a blood loss that exceeded the calculated maximum allowable blood loss for the patient (i.e., 100 mL). Therefore, initial resuscitation with warm replacement fluids (i.e., 1% glucose solution in an isotonic balanced salt solution) in 3:1 ratio (for 1 mL blood loss 3 mL fluid), was done. Followed by packed red blood cells transfusion in 1:1 ratio (for 1 mL blood loss 1 mL blood), and norepinephrine 0.01-0.08 mcg/kg/min was administered to maintain mean arterial pressure within an acceptable range (i.e., 20% of baseline).

GA was maintained with oxygen, air, and a sevoflurane mixture titrated to an age-adjusted minimum alveolar concentration (MACage) value of 0.9-1.0. Intraoperatively, blood gas analysis showed hypoxemia (PaO_2 _- 65.8 mmHg) and hypercarbia (PaCO_2_ - 61.1 mmHg) with acidosis (PH - 7.21). Therefore, the mechanical ventilatory settings were modified to optimize the gas parameters, but the hypoxemia and respiratory acidosis resolved only after the surgery when the baby was turned supine and ventilated.

**Figure 5:**
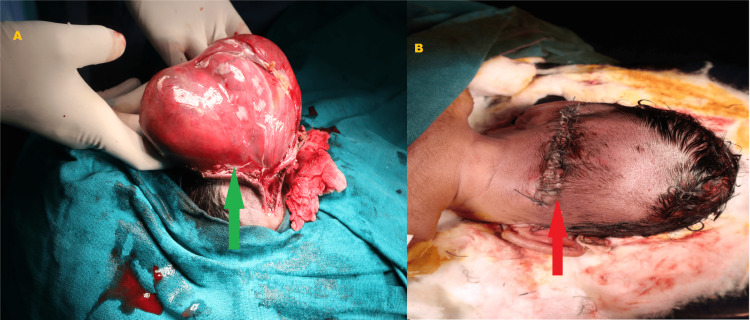
(A) Intraoperative photograph, green arrow showing the dissection of encephalocele sac. (B) Postoperative photograph, red arrow showing wound after repair.

During the surgery, an incision was given along the neck of the sac. The dissection was done layer by layer, and the sac and gliotic brain tissue were excised. Intraoperatively indocyanine green dye was used to confirm the presence of venous sinuses and blood vessels. They were dissected from the sac, preserved, and along with brain tissue, reposited back into the skull through the defect. Watertight dural closure was done. The subcutaneous layer was closed with vicryl 4-0, and skin closure was done with ethylene 3-0. The surgery lasted for almost six hours (Figures 5A, 5B).

The child was electively ventilated because of resection of the brain parenchyma, significant blood loss, prolonged duration of surgery, and ongoing vasopressor support. In the neonatal intensive care unit, the child was gradually weaned off the ventilator and extubated on the second postoperative day. Further, the child developed an infection at surgical site, resulting in wound dehiscence and brain abscess, treated with antibiotics as per culture sensitivity, wound resuturing and aspiration of abscess under GA, which prolonged the baby's overall hospital stay. Gradually, the child's general condition improved, with persistent residual weakness in both lower limbs. The child was discharged home two and a half months after surgery with a healthy wound and a plan for thoracic MMC repair later. The child has been followed up for the past six months and is doing well, but the lower limb strength has remained the same.

## Discussion

A midline defect of cranial bone fusion leading to encephalocele occurs most frequently in the occipital region. As the size grows, lying in the supine position becomes nearly impossible. Occasionally, encephalocele is associated with cerebral arterial and venous anomalies. Approximately 60% of these children may have related abnormalities affecting perioperative management, such as hydrocephalus, microcephaly, micrognathia, Chiari malformation, pulmonary hypoplasia, and renal agenesis [4]. MMC is the most common NTD, characterized by a defect in the vertebral column and skin with exposure to neural and meningeal elements. Encephalocele and MMC in the same patient have rarely been reported [3]. Mealey et al. observed that out of 623 neural tube abnormalities, only four patients had an encephalocele and an MMC [5].

The significant perioperative anesthetic challenges encountered in managing occipital encephalocele are maintaining adequate positioning of the neonate on the operating theatre table during induction and securing and maintaining the airway afterward. The anticipated difficult airway due to the presence of the mass makes this a formidable task. Improper positioning and limited neck extension can make mask ventilation and endotracheal intubation difficult or impossible. Neonates are susceptible to hypoxemia because of their low functional residual capacity, small closing capacity, high oxygen consumption, and increased risk of airway collapse, which worsens after the induction of GA, and they respond to hypoxemia with early bradycardia and even cardiac arrest [6].

Another concern with occipital encephalocele and thoracic MMC includes preventing excess pressure on the sac while achieving a neutral supine position of the head to secure the airway. Any excessive pressure on the sac can increase the intracranial pressure or cause the sac to rupture, resulting in sudden decompression. Intubation in the lateral position has long been used, as suggested by Creighton et al. and Jain et al., to reduce any pressure on the sac [7,8]. However, this position is unfamiliar for intubation. Further, a huge occipital encephalocele will also hamper the head extension required for laryngoscopy. Placing the child's head on the edge of the table with an assistant supporting the sac has also been used to achieve an optimal position for airway handling [9,10]. This technique involves the presence of an assistant at the head end of the table, which may create a space constraint. In another method described by Mowafi et al., a C-shaped gel foam doughnut was used along with sheets and pillows to form a platform, and the child was placed supine on it with the head resting on the gel foam doughnut and the swelling hanging freely [11]. Karim et al. concluded that placing the head along with the occipital encephalocele in an adjustable horseshoe headrest provided a viable adjunct for airway management in such cases [12]. In our case, we had to take care of two swellings. We used a technique similar to that described by Pahuja et al. [13]. We made stacks using surgical drapes and cotton rolls to create a flexible and adjustable platform for head placement and a padded depressed square space for occipital encephalocele support. Pahuja et al. used a pillow to support the torso, and we used stacks made up of bigger surgical drapes covered with cotton to support the same. With this arrangement, the thoracic MMC rested and supported on the "doughnut" ring cushion, made of cotton roll on a body part of the stacks. This provided us with optimal intubation conditions and eliminated the risk of compression-related preoperative sac rupture or an inadvertent increase in intracranial pressure. However, no one technique is demonstrably superior to another [12], and it always depends on the clinical presentation.

In such cases, an inhalational agent is preferred for induction, as it can be rapidly washed off during difficult circumstances, and apnoea is rare. Although the use of muscle relaxants is debatable, Davys et al. found that adding 0.3 mg/kg of rocuronium to 8% sevoflurane improved intubating conditions and decreased the incidence of adverse respiratory events (laryngospasm, bronchospasm, and SpO_2_ of below 90%). This finding suggests that skipping a muscle relaxant may, paradoxically, cause respiratory difficulties [14]. In such a situation, the attending anesthesiologist's judgment determines whether to use a muscle relaxant or not. In our patient, succinylcholine was used to intubate the child without triggering a hyperkalemic response after ensuring adequate mask ventilation [15].

CSF drainage with a needle under sterile precautions before induction is mentioned in some cases [16]. However, draining might deteriorate the patient's condition, and hamper cerebral perfusion; there is a risk of bleeding, infection, volume loss, electrolyte disturbances, and sudden cardiovascular arrest owing to the traction of cerebral neuronal pathways involving the brainstem nuclei [16].

Intraoperative hemodynamic challenges add another dimension to the management of these cases. The margin for blood loss in neonates is minuscule, and meticulous control is required while keeping in mind the adverse effects of blood transfusion. In neonates, the suboccipital bone is richly vascularized, and the dural sinuses are extensive and ill-defined [7]. In addition, dissection of the large encephalocele sac and blood vessels in the sac, in our case, complicated the surgery and led to intraoperative blood loss that exceeded the allowable limit, causing hemodynamic instability. Intensive monitoring, estimation of blood and fluid loss, meticulous replacement of loss with warm fluids, blood, and timely administration of vasopressor have helped us maintain stable hemodynamics during surgery.

Management of intraoperative respiratory complications is also challenging. The trachea is usually short in neonates, with a minimal safety margin for the endotracheal tube tip. The prone positioning and surgical handling of the occipital encephalocele can displace the tip, jeopardizing or complicating the gas exchange.

Latex allergies and sensitization have been well documented in patients with NTDs. The manifestation of an allergic reaction can range from intraoperative bronchospasm to sudden cardiorespiratory arrest [17]. Therefore, latex precautions, latex-free gloves, and masks should be preferred for these patients. We also used latex-free gloves.

In neonates with multiple NTDs, there is a greater risk of hypothermia in these patients including mortality [18]. Therefore, ambient operation theatre temperature (27 degrees Celsius), warming mattress, blood, and fluids through a fluid warmer, covering extremities with cotton blanket, heated and humidified anesthetic gases, and strict intraoperative temperature monitoring should be considered.

In addition to postoperative wound infection, sepsis, and brain abscess, a seizure is another crucial factor affecting the postoperative outcome of these patients. Mahajan et al. reported 17.2% seizures in patients with postoperative occipital encephalocele [16]. Therefore, prophylactic antiepileptic and antibacterial treatment postoperatively is required.

## Conclusions

Perioperative management of neonates with the simultaneous presence of giant vascular occipital encephalocele and thoracic MMC is challenging. An open mind for adapting the armamentarium, especially for airway management in context to the updated knowledge of potential difficulties during the perioperative period, is crucial. In addition, associated congenital anomalies, unusual intraoperative positioning, blood loss, hemodynamic instability, respiratory complication, electrolyte abnormalities, hypothermia, and cardiorespiratory disturbances increase the challenges manifold. Further turbulent postoperative care for different expected complications is also not uncommon. Although the prognosis is poor, a multidisciplinary approach, individualized care, vigilant monitoring, and timely recognition and treatment of complications can lead to successful perioperative management of such patients.
